# Teste de Esforço para Detecção de Vias Acessórias de Alto Risco em Pacientes com Síndrome de Wolff-Parkinson-White

**DOI:** 10.36660/abc.20250194

**Published:** 2025-05-06

**Authors:** Fabricio Vassallo

**Affiliations:** 1 Hospital Santa Casa de Vitória Faculdade de Medicina Vitória ES Brasil Faculdade de Medicina do Hospital Santa Casa de Vitória, Vitória, ES – Brasil; 2 Hospital Santa Rita Vitória ES Brasil Hospital Santa Rita, Vitória, ES – Brasil

**Keywords:** Síndrome de Wolff-Parkinson-White, Teste de Esforço, Técnicas e Procedimentos Diagnósticos

Na descrição de Wolff, Parkinson e White em 1930 da via acessória exibindo condução anterógrada, foi feito muito trabalho para descrever a anatomia, eletrofisiologia e história natural da síndrome.^1^ Durante o ritmo sinusal, um padrão típico no eletrocardiograma (ECG) de repouso está presente: um intervalo PR curto (< 120 ms), um movimento ascendente ou descendente arrastado do complexo QRS (conhecido como onda Delta) e um amplo Complexo QRS (>120ms). Na maioria dos casos, as vias acessórias que dão origem aos padrões de Wolff-Parkinson-White (WPW) são observadas em corações estruturalmente normais.^2^

Pacientes com síndrome de WPW apresentam palpitações causadas por taquicardia atrioventricular ou, menos comumente, por taquicardia atrial primária ou fibrilação.

A incidência de vias acessórias e pacientes com síndrome de WPW é descrita em 1 a 3 pacientes em 1000 indivíduos, e naqueles com parente de primeiro grau com essa situação é descrita como sendo de 5,5 em 1000. Atualmente, a descrição de indivíduos assintomáticos com ECG de repouso com padrão de WPW é de 65% em adolescentes e 40% naqueles com mais de 30 anos.^3,4^ Pacientes com WPW também são vulneráveis a arritmias fatais devido à fibrilação atrial pré-excitada, levando à fibrilação ventricular e morte súbita cardíaca (MSC). Embora a incidência de MSC, segundo estudos observacionais, seja muito baixa, foi descrita uma incidência de 4,5 episódios de morte súbita por 1000 pacientes-ano em adultos assintomáticos seguidos por mais de 3 anos.^5^ A possibilidade de MSC no padrão de ECG WPW é a maior preocupação quando estamos cara a cara com esses pacientes, e a razão para a estratificação de risco da via acessória. Em sua forma mais simples, a estratificação de risco da via acessória é uma avaliação não invasiva que utiliza basicamente monitoramento Holter de 24 horas e teste de esforço. De acordo com os resultados desses dois exames e diante da incapacidade de provar uma perda abrupta absoluta clara de pré-excitação manifesta, a consideração de uma estratificação invasiva é necessária. Outro grupo de pacientes que tem indicação formal para estratificação invasiva com estudo eletrofisiológico (EEF), apesar da ausência de sintomas, é o grupo de alto risco de ocupações profissionais e atletas competitivos. O objetivo do EEF é avaliar e determinar o menor período refratário efetivo anterógrado (PREA) da via acessória. Quanto menor o PREA, maior o risco de desenvolver uma arritmia letal. O PREA definido na identificação de pacientes de alto risco é ≤ 250 ms. Se uma fibrilação atrial pré-excitada for induzida durante o teste EP, outra medida pode ser usada que inclua o menor intervalo RR pré-excitado (SPERRI). Os pacientes de alto risco também são identificados com um SPERRI ≤ 250 ms.

Nesta edição do ABC Cardiol, Alencar et al.^6^ publicou uma metanálise da precisão do teste de esforço na detecção de vias acessórias de alto risco. O objetivo do estudo foi avaliar a precisão diagnóstica de testes de esforço não invasivos em comparação com EEF na identificação de vias acessórias de alto risco na síndrome de WPW. De um total de 309 estudos selecionados, 6 preencheram os critérios de elegibilidade para análise, incluindo um total de 765 pacientes. Usando o mesmo entendimento de que pacientes de menor risco durante os testes de esforço são aqueles com perda da medição de pré-excitação. E para pacientes de alto risco, durante EEF, um PREA e SPERRI de ≤ 250 ms. Seus resultados mostraram que o valor da razão de verossimilhança negativa (LR-) é 0,260. Isso significa que após um resultado negativo no teste de esforço, a probabilidade da presença de uma via acessória de alto risco no EEF é aproximadamente 4 vezes menor. Em outras palavras, um teste negativo reduz significativamente a chance de o paciente ter uma via acessória de alto risco. Além disso, a análise de sensibilidade, que foi limitada a pacientes pediátricos, mostrou resultados consistentes, indicando que essa conclusão também é válida para a população pediátrica.

Em sua discussão, eles recomendam que o EEF seja o padrão ouro para estratificação de risco em pacientes WPW. Eles argumentam que, apesar de reduzir a probabilidade de uma via de alto risco por um fator de 4, o que obviamente é uma descoberta muito importante, eles acreditam que essa redução por si só não é suficiente para estabelecer o teste de estresse por exercício como uma ferramenta definitiva para estratificar vias acessórias de alto ou baixo risco.

De acordo com a *ESC Task Force* de 2019 para o gerenciamento de pacientes com taquicardia supraventricular, as indicações, de maior a menor evidência, em pacientes com vias acessórias assintomáticas são: A) EEF para profissões de alto risco e atletas competitivos; B) EEF para estratificação de risco para todos os pacientes e C) estratificação de risco não invasiva para todos os pacientes.^7-9^ Mesmo estudos invasivos não conferem certeza absoluta sobre pacientes de alto risco. Em um estudo retrospectivo com 912 pacientes jovens (≤ 21 anos) com síndrome de WPW, 96 (10,5%) apresentaram eventos com risco de vida, dos quais 49% apresentaram FA pré-excitada rapidamente conduzida. Para aqueles pacientes com eventos e submetidos a EEF, 22 de 60 (37%) não apresentaram características de alto risco determinadas por EEF, e 15 de 60 (25%) não apresentaram características de via preocupantes nem taquicardia supraventricular induzível.^10^

Como a EEF e a ablação por cateter para estratificação e tratamento da síndrome de WPW, quando realizadas por um operador experiente, estão associadas a uma alta taxa de cura (>95%) e baixo risco (<0,5%) de complicações graves, elas devem ser o padrão ouro para o tratamento desses pacientes.
