# Supplementary material for: Teste de Esforço para Detecção de Vias Acessórias de Alto Risco em Pacientes com Síndrome de Wolff-Parkinson-White
Source: Arq Bras Cardiol. 2025 May 6;122(4):e20250194. [Article in Portuguese] doi: 10.36660/abc.20250194 (PMC12097719; doi:10.36660/abc.20250194)
Supplement: Translation

# Translation: Exercise Stress Test For Detecting High-Risk Accessory Pathways In Patients With Wolff-Parkinson-White Syndrome

**DOI:** 10.36660/abc.20250194i

**Authors:** Fabricio Vassallo

**Affiliations:** 1 Faculdade de Medicina do Hospital Santa Casa de Vitória Vitória ES Brazil Faculdade de Medicina do Hospital Santa Casa de Vitória, Vitória, ES – Brazil; 2 Hospital Santa Rita Vitória ES Brazil Hospital Santa Rita, Vitória, ES – Brazil

**Keywords:** Wolff-Parkinson-White Syndrome, Exercise Test, Diagnostic Techniques and Procedures

In the description of Wolff, Parkinson, and White in 1930 of accessory pathway exhibiting antegrade conduction much work had been done to describe the anatomy, electrophysiology, and natural history of the syndrome.^1^ During sinus rhythm, a typical pattern in the resting electrocardiogram (ECG) is present: a short PR interval (< 120ms), a slurred upstroke or downstroke of the QRS complex (known as Delta wave), and a wide QRS complex (>120ms). In most cases, accessory pathways giving rise to the WPW patterns are seen in structurally normal hearts.^2^

Patients with WPW syndrome presented with palpitations caused by an atrioventricular tachycardia or, less commonly, due to primary atrial tachycardia or fibrillation.

Incidence of accessory pathways and WPW syndrome patients are described in 1 to 3 patients in 1000 individuals, and in those with a first-degree relative with this situation is described to be 5.5 in 1000. At present, the description of asymptomatic individuals with a resting ECG with WPW pattern is 65% in adolescents and 40% in those with more than 30 years.^3,4^ Patients with WPW are also vulnerable to fatal arrhythmias due to pre-excited atrial fibrillation, leading to ventricular fibrillation and sudden cardiac death (SCD). Although the incidence of SCD, according to observational studies, is very low, an incidence of 4.5 episodes of sudden death per 1000 patients-years was described in asymptomatic adults followed by more than 3 years.^5^ The possibility of SCD in the WPW ECG pattern is the highest concern when we are face to face with these patients and the reason for the risk stratification of the accessory pathway. In its simplest form, risk stratification of the accessory pathway is a non-invasive evaluation utilizing basically 24-hour Holter monitoring and exercise stress test. According to the results of these two exams and facing the inability to prove a clear absolute abrupt loss of manifest preexcitation, consideration for an invasive stratification is necessary. Another group of patients who have a formal indication for invasive stratification with electrophysiological study (EPS), despite the absence of symptoms, is the high-risk group of professional occupations and competitive athletes. The objective of the EPS is to evaluate and determine the shortest antegrade effective refractory period (APERP) of the accessory pathway. The lower the APERP, the higher the risk of developing a lethal arrhythmia. The defined APERP in the identification of high-risk patients is ≤ 250ms. If a pre-excited atrial fibrillation is induced during the EP test, another measurement can be used that includes the shortest pre-excited RR interval (SPERRI). The high-risk patients are also identified with a SPERRI ≤ 250ms.

In this issue of ABC Cardiol, Alencar et al.^6^ published a meta-analysis of the accuracy of exercise stress test in the detection of high-risk accessory pathways. The objective of the study was to evaluate the diagnostic accuracy of non-invasive exercise tests in comparison to EPS in the identification of high-risk accessory pathways in WPW syndrome. Of a total of 309 screened studies, 6 fulfilled the eligibility criteria for analysis, including a total of 765 patients. Using the same understanding that lower-risk patients during exercise stress tests are those with the loss of preexcitation measurement. And for high-risk patients, during EPS, an APERP and SPERRI of ≤ 250ms. Their results showed that the negative likelihood ratio (LR-) value is 0.260. This means that after a negative result on the exercise stress test, the probability of the presence of a high-risk accessory pathway in EPS is approximately 4 times lower. In other words, a negative test significantly reduces the chance that the patient will have a high-risk accessory pathway. In addition, the sensitivity analysis, which was limited to pediatric patients, showed consistent results, indicating that this conclusion is also valid for the pediatric population.

In their discussion, they recommend that EPS should be the gold standard for risk stratification in WPW patients. They argue that despite reducing the likelihood of a high-risk pathway by a factor of 4, which obviously is a very important finding, they believe this reduction alone is not sufficient to establish exercise stress test as a definitive tool for stratifying high- or low-risk accessory pathways.

According to the 2019 ESC Task Force for the management of patients with supraventricular tachycardia, the indications, from higher to low evidence, in asymptomatic accessory pathways patients are: A) EPS for high-risk professions and competitive athletes; B) EPS for risk stratification for all patients and C) non-invasive risk stratification for all patients.^7-9^ Even invasive studies do not confer absolute certainty about high-risk patients. In a retrospective study with 912 young patients (≤ 21 years) with WPW syndrome, 96 (10.5%) experienced life-threatening events, of whom 49% had rapidly conducted pre-excited AF. For those patients with events and subjected to EPS, 22 of 60 (37%) did not have EPS-determined high-risk characteristics, and 15 of 60 (25%) had neither concerning pathway characteristics nor inducible supraventricular tachycardia.^10^

Since the EPS and catheter ablation for stratification and treatment of WPW syndrome, when performed by an experienced operator, is associated with a high cure rate (>95%) and low risk (<0.5%) of major complications, it should be the gold standard for managing those patients.
